# A Sexual Health Self-Management Intervention (Psychosexual Educational Partners Program) for Couples With a History of Breast and Gynecological Cancer: Mixed Methods Feasibility Study

**DOI:** 10.2196/75743

**Published:** 2025-10-21

**Authors:** Noël M Arring, Jennifer Barsky Reese, Carolyn Lafferty, Debra L Barton, Jeanne Carter

**Affiliations:** 1 College of Nursing University of Tennessee at Knoxville Knoxville, TN United States; 2 Cancer Prevention and Control Program Fox Chase Cancer Center Philadelphia, PA United States; 3 Gynecology Service Department of Surgery and Department of Psychiatry & Behavioral Sciences Memorial Sloan Kettering Cancer Center New York, NY United States

**Keywords:** breast cancer, cancer, couples-based, dyad, gynecological cancer, oncology, partners, self-management, sexual communication, sexual health

## Abstract

**Background:**

Women with breast or gynecological cancer and their intimate partners often face sexual problems in their relationships. Accessing care for sexual health problems is challenging for several reasons (eg, limited trained health care providers and privacy concerns), making self-management approaches highly promising.

**Objective:**

This study assessed the feasibility of the Psychosexual Educational Partners Program (PEPP), a 6-week sexual health self-management intervention for women treated for breast or gynecological cancer and their intimate partners.

**Methods:**

A mixed methods single-arm, repeated measures design was used for this study. An attrition rate of ≤25% was considered feasible. Intervention experiences were assessed via interviews, and preliminary effects on the Dyadic Sexual Communication Scale, relationship quality measured by the Revised Dyadic Adjustment Scale, and sexual health measured by PROMIS Sexual Function and Satisfaction version 2.0 were explored quantitatively.

**Results:**

In total, 7 (77%) of the 9 couples completed the study through week 6 and provided both pre- and poststudy data, resulting in an attrition rate of 22% (2/9), which met the feasibility benchmark for attrition of 25% or less. The following two themes emerged: (1) PEPP *helped us start difficult conversations* and impacted *emotional and physical intimacy*. The intervention adherence was 85%. Dyadic Sexual Communication Scale scores improved with a mean change score of 6.64 (SD 9.65) and a Cohen *d* of 0.69. Revised Dyadic Adjustment Scale scores declined slightly, with a mean change score of –0.93 (SD 3.41) and a Cohen *d* of 0.27. PROMIS Sexual Function and Satisfaction version 2.0 scores showed small improvements for women on desire, with a mean change score of 2.36 (SD 6.24) and a Cohen *d* of 0.38. Similarly, for women, the satisfaction mean change score was 2.20 (SD 8.22) and a Cohen *d* of 0.27. For intimate partners, a small effect was found for desire, but in this instance, desire decreased with a mean change score of –1.57 (SD 6.09) and a Cohen *d* of 0.26.

**Conclusions:**

The findings support PEPP as a feasible intervention for improving sexual communication. If proven effective in a randomized controlled trial, it has the potential to address the critical gap in supportive care among female cancer survivors.

**Trial Registration:**

ClinicalTrials.gov NCT05070299; https://clinicaltrials.gov/study/NCT05070299

## Introduction

### Background

As many as 90% of women with a history of gynecological cancer and 70% of women with a history of breast cancer report issues with sexual health [1]. These concerns encompass both negative physical symptoms and a woman’s cognitive and emotional sexual connection [1-3]. Changes in intimacy include sexual communication, sexual interest and satisfaction [2], and one’s appearance or body and self-image [3].

In addition, partners of cancer survivors often experience distress related to cancer-related sexual problems, as well as negative effects on their own sexual functioning [4-6]. It has long been recognized that the quality of a partnered relationship is a significant predictor of a woman’s sexual health after breast cancer [2,7]. To understand the individual and interdependent experiences of female cancer survivors and their partners, it is critical to examine how such couples navigate their experiences within the context of their relationships [8,9]. According to the theory of dyadic illness management [10], patients and their partners navigate the illness together. For couples facing life after cancer, the challenges can be both physical and psychological [10,11] in nature. Recent systematic reviews highlight the efficacy of a couple-based approach for improving relationship dynamics in the presence of adequate dyadic coping after cancer [12-14]. A couple-based approach is also consistent with the 2017 American Society of Clinical Oncology sexual health guidelines, which call for health care providers to include the partner if the patient wishes [15].

Access to couple-based psychosexual interventions is often restricted due to the limited number of health care providers equipped to deliver these interventions. Therefore, self-management approaches hold promise for reaching a greater number of patients while enhancing the cost-effectiveness, flexibility, and accessibility of the intervention. In addition, self-management strategies for sexual health among cancer survivors are promising [16-18]. On the basis of our previous research and clinical data from an established female sexual medicine program [15], our interdisciplinary team developed the Psychosexual Educational Partners Program (PEPP), a self-managed intervention.

### Objectives

The primary aim of this study was to assess the feasibility of the PEPP, a 6-week sexual health self-management intervention for couples and women treated for breast or gynecological cancer who report cancer-related sexual changes and their intimate partners. We selected an attrition rate of 25% or less as our *a priori* feasibility benchmark. In addition, the study evaluated participants’ intervention experiences using a qualitative investigation and explored the impact of the intervention on sexual communication, relationship quality, and sexual health.

## Methods

### Study Design

This feasibility study of the PEPP, a sexual health self-management intervention for couples, included a mixed methods single-arm, repeated measures design.

### Ethical Considerations

This study was approved by the University of Michigan Institutional Review Board (HUM00202063) and registered on ClinicalTrials.gov (NCT05070299). All participants provided written informed consent between March and July 2022. All study data were deidentified before analysis. Compensation for study participation was up to US $50 per person or US $100 per couple.

### Setting and Recruitment Procedures

This study was conducted at the University of Michigan, a National Cancer Institute–designated comprehensive cancer center located in Ann Arbor, Michigan. Recruitment involved physician referrals, the University of Michigan Health research portal (a clinical trial volunteer database), cancer registry mailings, and community outreach (eg, flyers and presentations). Once a potential participant reached out to the study team, their eligibility was assessed using a checklist derived from the inclusion and exclusion criteria described in the following sections. Women who met the eligibility requirements were sent information about the study’s purpose, procedures, and expectations, along with their partners. If both expressed interest in participating, they were sent a consent form and scheduled for a consent visit. Consent visits were conducted via a secure Health Insurance Portability and Accountability Act–compliant video platform or by phone. Consent was obtained using an institutional review board–approved electronic system during this session. Once consent was obtained, the participants were sent the PEPP workbook and scheduled for their baseline visit.

### Participants

The study participants were enrolled as couples, consisting of women who had been treated for breast or gynecological cancer and their intimate partners. Eligible participants were females with a history (any stage) of breast or gynecological cancer aged ≥18 years who had completed primary treatment (chemotherapy, radiation, or surgery) ≥3 months and ≤5 years before registration, reported that they had experienced a negative change in communication or intimacy since their diagnosis or treatment, and were able to read and write in English. Participants must also have had a stable partner, defined as anyone with whom the woman has had an intimate relationship for at least 3 months before her cancer diagnosis. Concurrent adjuvant endocrine therapy or HER2-targeted therapy was allowed. Exclusion criteria included individuals on an antidepressant for less than 30 days before registration, or with an unstable dose (expected change in dose or treatment); a history of sexual abuse; a psychiatric disorder such as major depressive disorder, bipolar disorder, obsessive compulsive disorder, or schizophrenia; or enrollment in another study addressing sexual health.

### Intervention

PEPP, a sexual health self-management intervention for couples, was developed by a multidisciplinary team and informed by clinical data from an established female sexual medicine program [15]. PEPP included a self-administered workbook with 3 chapters normalizing intimacy after cancer, reconnecting, and expanding communication and intimacy (Table 1) and appendices written at a reading level of grade 6.5. Phone call check-ins were completed at the end of each module with at least one member to assess the couples’ adherence to the intervention components, identify adverse events, and address questions or concerns. Couples were instructed to complete one chapter every 2 weeks but were allotted additional time if needed.

**Table 1:** Workbook chapters included in the Psychosexual Educational Partners Program.

Title	Content	Goals
Chapter 1: Normalizing intimacy after cancer	Physical and emotional sequelae of cancer treatment Identifying challenges and barriers Positive thinking and unhelpful thoughts Mindfulness exercise, including progressive muscle relaxation	Improved understanding of sexual health challenges created by cancer and cancer treatment Improved awareness of challenges (physical, emotional, and communication) experienced by partner Improved mindfulness
Chapter 2: Reconnecting	Answering “What is Mindfulness?” Sensate focus 101 Goal-setting activities	Increased awareness about ways to approach physical intimacy Practical skills for goal setting as a couple
Chapter 3: Expanding communication and intimacy	Communication 101 Mindfulness, including guided imagery of leaves on a stream activity	Increased knowledge of verbal and nonverbal communication

The PEPP workbook was written as a motivating, nonjudgmental guide providing information to support couples as they work toward rebuilding intimacy after cancer and creating healthy behavioral changes together. To enhance couples’ motivation, 4 key components of motivational interviewing were integrated into the workbook: partnership, acceptance, compassion, and evocation [19]. First, the workbook begins with an overview of sexual health after cancer and then acknowledges a variety of physical and emotional challenges that couples often face. The goal is to help couples engage with the material according to their level of readiness and comfort (partnership). Couples are routinely encouraged to modify their activities to suit their preferences and needs (acceptance). The couples’ experiences, interests, and needs are prioritized when they are asked questions or presented with activities (compassion). Finally, through carefully crafted *PEPP talks* and activities, the workbook provides a vehicle for couples to think about what is important to them and articulate their own steps toward behavior change (evocation).

### Data Collection

Following the informed consent process, each participant completed a demographic survey and the Dyadic Sexual Communication Scale (DSC), Revised Dyadic Adjustment Scale (RDAS), and PROMIS Sexual Function and Satisfaction version 2.0 (PROMIS SexFS). Data were collected using the REDCap (Research Electronic Data Capture; Vanderbilt University) at baseline and week 6. Participant experience interviews were conducted using the Health Insurance Portability and Accountability Act–compliant Zoom (Zoom Video Communications) within 14 days of workbook completion.

### Study Measures

Attrition was defined as any participant who did not provide data for both pre- and postquantitative measures. Participants were considered to have completed the study if they provided quantitative and qualitative data in week 6. Our *a priori* benchmark was that an attrition rate of 25% or less would be considered feasible [20].

To evaluate the participants’ experiences with the intervention, each couple participated in an interview within 14 days at the end of week 6. The interview questions were based on the experience of the research team. Questions prompted responses ranging from simple feedback on the length of the intervention to more complex input about its impact. Questions were developed to assess participants’ experiences with the workbook, such as perceived usefulness, time commitment, and relevance of the content.

The DSC is a 13-item instrument that assesses sexual communication between partners. The revised scale uses a 5-point response option where 3 is neutral [21]. Items were reverse scored so that high scores reflected more positive communication related to their sexual relationship. Possible scores range from 13 to 65, with higher scores representing a perception of better sexual communication with their partners. The original scale discriminated between couples with and without sexual problems, with a Cronbach α of 0.87 [22].

The RDAS is a 14-item scale with 3 themes (satisfaction, cohesion, and consensus) that measures an individual’s perception of the quality of a relationship with an intimate partner using a 6-point Likert scale [23]. The scores range from 0 to 69. Higher scores indicate more positive dyadic adjustment. Scores ≤47 indicate relationship distress. This scale was found to have high reliability, with a Cronbach α of 0.90 [23].

PROMIS SexFS V2 measures sexual activities, symptoms, functioning, and evaluation of sexual experiences [24]. General screener items ask about sexual activity and reasons for not engaging in sexual activity in the past 30 days. Female participants answered the 14-item version, and male participants answered the 10-item version. On both instruments, the desire and satisfaction subscales each included 2 items, and raw scores for each subscale ranged from 2 to 10. Raw scores for the desire and satisfaction domains were converted to T-scores using PROMIS T-score look-up tables, with a mean of 50 (SD 10) in the referent population. Higher scores indicate more of the domains being assessed. In the validation study, Cronbach α scores ranged from 0.85 to 0.98 [25]. This measure has been used in other studies on dyadic sexual health interventions with similar reliability and validity [26].

### Data Analysis

All demographic and outcome variables were summarized using descriptive statistics (mean, SD, percentage, and frequency). To evaluate the intervention’s impact on sexual communication, relationship quality, and sexual health, effect sizes were calculated with Cohen *d* from baseline to week 6. Means, variance, 95% CIs, and effect sizes were calculated to better estimate what sample size is needed for a larger trial with at least 80% power to detect at least a medium effect size. PROMIS domain scores can only be calculated using lookup tables when participants respond to all items in the domain. Therefore, missing data on the PROMIS scale resulted in participants being dropped from the analyses. SPSS statistical software (version 27; IBM Corp) was used for the analyses.

Participant interviews were conducted by one study team member (CL) to evaluate the intervention and the next steps in its delivery. The interviewer was known to the participants through recruitment and biweekly phone check-ins. Qualitative data were deidentified and summarized using interview questions. In total, 12 items prompted simple feedback that was quantified, and descriptive statistics were summarized. Three open-ended items could be described as “what” questions [27], such as “What, if any, impact has the workbook made on your ability to communicate with one another about difficult topics?” To respond to these exploratory items, a qualitative description approach was adopted, and content analysis was conducted to examine the 3 open-ended questions [27]. Two study team members (NMA and CL) independently coded the data with a focus on straight description, where coding remained consistent with the language used by participants [28]. The codes were reviewed by the coding team, and discrepancies were resolved through consensus.

## Results

### Sample Demographics

**Figure 1:**
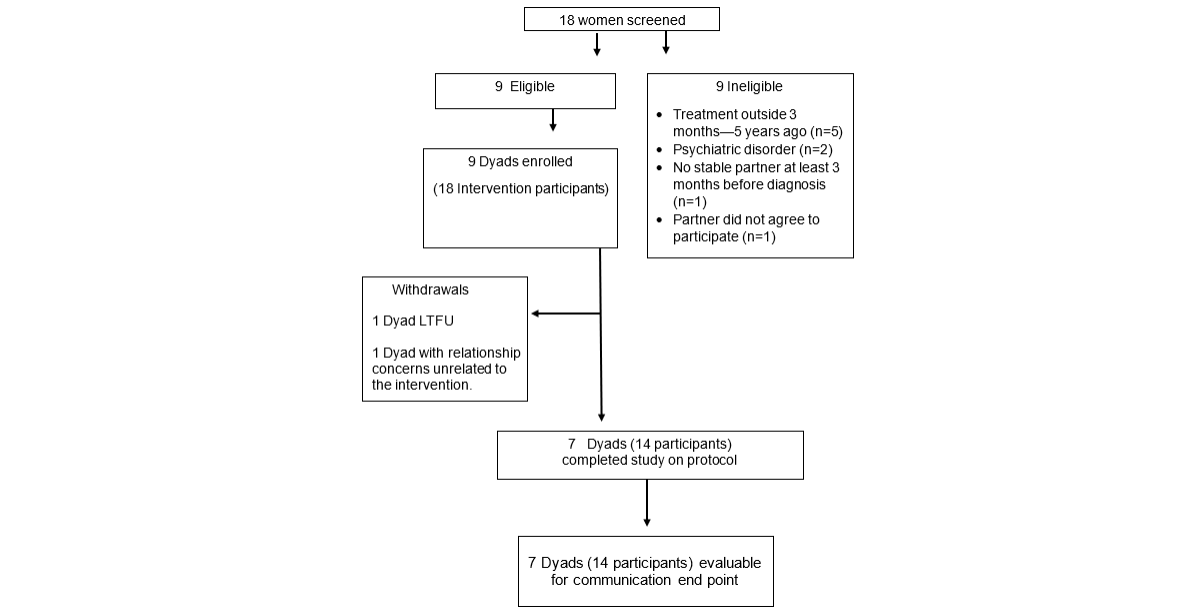
CONSORT (Consolidated Standards of Reporting Trials) diagram of the Psychosexual Educational Partners Program. LTFU: loss to follow-up.

In total, 9 couples, including 1 gynecological and 8 breast cancer survivors, were recruited for 18 participants (Figure 1). Participant ages ranged from 30 to 75 years; 17 (94%) identified as White, 1 (6%) as Hispanic; 15 (83%) reported a college degree or higher; 14 (78%) reported an income of US $100,000 or more; and 8 (89%) couples were married.

### Feasibility

In total, 7 (77%) of the 9 couples, for 14 participants, completed the study through week 6 and provided both pre- and poststudy data, resulting in an attrition rate of 22% (2/9) of couples, which met the feasibility benchmark for attrition of 25% or less. Of the 2 (22%) couples who did not complete the study, 1 (11%) couple withdrew in week 4 to seek couples therapy for concerns unrelated to the intervention, the other was lost to follow-up, and one couple included the study’s only gynecological cancer survivor.

The 7 (77%) couples who completed the study read the content in the 3 chapters. There was an 85% completion rate for the 17 activities included in the workbook. In addition, 2 (29%) of the 7 couples completed less than 50% of the activities. Moreover, 5 (71%) of the 7 couples completed all 17 activities.

### Participant Experience: Qualitative Description

Codes based on participant language and supporting quotations are provided in Table 2, and a full qualitative analysis is presented in Multimedia Appendix 1. The code “helped us start difficult conversations” emerged in response to 2 of the 3 questions. Overall, half (7/14, 50%) of the participants reported that the 6-week time frame was just right. In total, 8 (57%) of the 14 participants stated that it was a helpful guide for discussions.

**Table 2:** Qualitative description of the Psychosexual Educational Partners Program: open-ended questions, themes, and supporting quotes (N=14).

Questions	Answers
Question 1: How (or in what way) did the workbook match your understanding of the sexual health challenges created by cancer and cancer treatments?	It was normalizing (n=2, 14%) “I’m not alone in this. This is a common thing. it’s not like my fault or our fault that...we have these challenges, but...this is like a normal issue.” [Survivor] “It helped me to understand that what we were experiencing was not just normal, but actually pretty common...I wish I’d known this before. It would have just been helpful to know that lots of women, lots of couples go through this.” [Survivor] It gave us a new way to think about sexual health (n=3,21%) “I didn’t think about...you know, that bullet point had been affected by...cancer and treatment and things like that. So there were some things that I was like. ‘Oh, yeah that that makes sense that I hadn’t thought about before.’” [Survivor] “[M]aking sure intimacy is defined not just as a goal to sexual intercourse, but a goal to physical [and] emotional connection.” [Partner] Helped us start difficult conversations (n=3, 21%) “Where it was tremendously useful was in getting us to...discuss things that I didn’t want to discuss. It’s more me, [my partner] will talk about anything, certainly to do with sex and I have been very reticent and this was really forcing me to. We talked about things.” [Survivor]
Question 2: What, if any, impact has the workbook made on your ability to communicate with one another about difficult topics?	Helped us start difficult conversations (n=2, 14%) “Before there was kind of [an] elephant in the room, and this...forced us to talk about it.” [Survivor] Opportunities for practice (n=6, 43%) “I think...the frequency of the conversations just sort of lends itself to just a closer connection and communication when we’re doing it on a regular basis.”[Survivor] Communication tools (n=6, 43%) “I statements have been really helpful...So it’s been helpful to just like take a second to pause and reframe what I want to say to him.” [Survivor] “I think that we have spent a lot more time communicating and kind of maybe pausing and reflecting on each other’s experience, and just like thinking about it from both perspectives.”[Partner]
Question 3: What, if any, impact has the workbook made in the level of intimacy of your relationship?	Emotional and physical intimacy (n=5, 36%) “The emotional intimacy piece...has like really facilitated some of the physical intimacy things as well, and so they’re kind of hand in hand.” [Partner] “[H]aving those discussions in and of itself is sort of an intimate experience.” [Survivor] Helpful (n=4, 29%) “I think we’re headed in the right direction, and that was super helpful as well.” [Survivor] “It was helpful. I think it was helpful.” [Partner]

### Sexual Health Outcomes

The DSC improved over the 6 weeks of the study (Table 3). Possible scores for these items ranged from 1 to 5, where higher scores indicated greater agreement. The overall mean score at baseline was 45.00 (SD 11.06), and at 6 weeks it was 51.64 (SD 11.71), with a mean change score of 6.64 (SD 9.65) and a Cohen *d* of 0.69.

**Table 3:** Sexual health outcomes of the Psychosexual Educational Partners Program at baseline and week 6.

					Values, mean (SD)			Values, mean change (SD)			Cohen *d*				
**Dyadic Sexual Communication Scale (n=14)**															
	Baseline				45.00 (11.06)			—^a^			—				
	6 wk				51.64 (11.71)			6.64 (9.65)			0.69				
**Revised Dyadic Adjustment Scale (n=14)**															
	Baseline				51.14 (6.32)			—			—				
	6 wk				50.21 (6.78)			−0.93 (3.41)			0.27				
**PROMIS Sexual Function and Satisfaction version 2.0**															
	**Women**														
		**Desire (n=7)**													
			Baseline				42.31 (9.87)			—			—		
			6 wk				44.67 (8.62)			2.36 (6.24)			0.38		
		**Satisfaction (n=6)**													
			Baseline				41.60 (3.30)			—			—		
			6 wk				43.80 (7.75)			2.20 (8.22)			0.27		
	**Partners**														
		**Desire (n=7)**													
				Baseline		55.63 (8.61)			—			—			
				6 wk		54.06 (9.71)			−1.57 (6.09)			0.26			
		**Satisfaction (n=6)**													
			Baseline				44.63 (6.90)			—			—		
			6 wk				45.60 (4.95)			0.97 (6.92)			0.14		

^a^Not applicable.

The RDAS scores declined slightly, with a mean score at baseline of 51.14 (SD 6.32), and at 6 weeks it was 50.21 (SD 6.78), with a mean change score of –0.93 (SD 3.41) and a Cohen *d* of 0.27. The 6-week score was highly correlated with the baseline score of 0.867 (*P*<.001).

The PROMIS SexFS scores showed small improvements for women for desire and satisfaction. The mean score for desire at baseline for the women was 42.31 (SD 9.87), and at 6 weeks it was 44.67 (SD 8.62), with a mean change score of 2.36 (SD 6.24) and a Cohen *d* of 0.38. The mean score for satisfaction at baseline for the women was 41.60 (SD 3.30), and at 6 weeks it was 43.80 (SD 7.75), with a mean change score of 2.20 (SD 8.22) and a Cohen *d* of 0.27. For partners, a small effect was found for desire, but in this instance, desire decreased from 55.63 (SD 8.61) at baseline to 54.06 (SD 9.71) at 6 weeks, reflecting a mean change score of –1.57 (SD 6.09) and a Cohen *d* of 0.26.

## Discussion

### Principal Findings

Overall, there was a 77% retention rate and 85% intervention adherence, which supports the feasibility of PEPP, a novel sexual health self-management intervention for couples surviving breast and gynecological cancer. Preliminary efficacy analyses indicate that the intervention yielded a medium effect on sexual communication, as measured by the DSC scale (*d*=0.69). In addition, among the women, small effect sizes were observed on the PROMIS SexFS subscales for desire (*d*=0.38) and satisfaction (*d*=0.27). These findings support the feasibility and potential efficacy of PEPP in enhancing sexual health outcomes among breast cancer survivors and their intimate partners.

The intervention was well received by the participants, with a retention rate of 77% (7/9), which surpassed the benchmark. These findings are consistent with those found in a recent systematic review of 19 trials of couple-based intervention studies by Li et al [13] and contribute to the literature by demonstrating the positive reception of a sexual health self-management intervention, whereas most previous interventions in this vein involved a trained facilitator [13]. Using a self-management approach could also be appealing to couples because it offers privacy when seeking help for sexual problems, a sensitive issue that some couples may feel uncomfortable discussing with others outside the relationship [29]. Some self-management interventions for sexual health among cancer survivors have shown promise but have been limited by high attrition [16-18]. This study used a low-touch (every 2-week phone check-in) approach, which we believe influenced our retention rate. Other studies addressing sexual or psychosocial concerns among cancer survivors who used a similar approach reported high retention rates [30-32].

Findings from the qualitative interviews identified specific ways in which couples perceived the self-management intervention as helpful in promoting sexual communication and intimacy. On the basis of participant feedback, it is recommended that PEPP be updated by shifting some of the activities of chapters 2-3 and updating some of the imagery used. The quantitative results are promising and suggest that workbook components designed to improve couples’ coping skills in overcoming communication barriers may have been effective, although the findings are preliminary. Taken together, these findings support the need for further research into the effectiveness of the PEPP intervention for improving sexual health communication among couples learning to rebuild intimacy after cancer.

Surprisingly, despite some promising increases in sexual communication and small improvements in desire among women and satisfaction among both groups on the PROMIS SexFS, we found that scores on the RDAS declined, with a small effect found for this change. Couples were in the nondistressed range on the RDAS at baseline and at 6 weeks. This small decline, as well as the small decrease in desire reported by partners on the PROMIS SexFS measure, may be a result of discussing sexual issues, which can be challenging for many couples. Because the couples included in the study were in nondistressed relationships, it could be that relationship quality may not change over the course of a 6-week study. It would be interesting to examine whether baseline relationship quality could serve as a moderator of the effects on sexual outcomes in future studies.

### Clinical Implications

Sexual health issues are often an unmet need for many women with a history of cancer. This was a small single-arm feasibility study of PEPP, a sexual health self-management intervention for couples. The PEPP intervention appears to be a promising and potentially easily disseminated self-management intervention; however, additional testing is needed to assess its efficacy. If found to be effective, it could serve as a tangible tool that health care providers could provide to patients and their partners to help them discuss and manage sensitive issues in their sexual relationships. While research is needed to assess its efficacy, it is vitally important for clinicians to initiate conversations about sexual health to guide patients to currently available resources.

### Research Implications

While the phone call check-ins at the end of each module provided a low-touch method for staying in contact with participants, recent research exploring self-management interventions supports the use of text or email reminders [33-36] or weekly assessments [37], which could improve the scalability of a self-managed intervention such as PEPP. Future research should continue to evaluate the most effective way to continue to engage participants in self-managed interventions and identify predictors for which couples are best served by self-managed couples’ interventions.

A major advantage of self-management interventions is the participation of couples who would not otherwise join due to discomfort in discussing sexual issues with a person outside the relationship or have time constraints limiting their ability to travel to seek help. However, the couples who participated in this study were likely to be highly intrinsically motivated; therefore, the data may not be generalizable to all couples with intimacy concerns following breast or gynecological cancer. Future research should assess potential moderators, including sexual distress, dyadic adjustment, and relationship quality.

### Study Limitations

As this was a feasibility study with a small sample size, there was limited ability to generalize these findings or determine the efficacy of the intervention, as there was no control condition or comparison group. Furthermore, the sample was largely White, well-educated, and of high socioeconomic strata. In addition, the final sample included only breast cancer survivors, which could signify that this may not be feasible for gynecological cancer survivors. Another potential limitation of this study is that the women were only eligible if their partners agreed to participate with them. Therefore, women with partners who are less supportive may be underrepresented.

### Conclusions

The findings support the potential benefit of the PEPP intervention in helping couples work to re-establish intimacy after breast and gynecological cancer and cancer treatment. Research is needed in a larger, more diverse population to evaluate the efficacy of the PEPP intervention compared to a control condition, to better understand its impact on sexual communication, and to test strategies that can support the wider dissemination of such an intervention.

## Appendix

Multimedia Appendix 1 Descriptive summaries of participant experiences.

## Abbreviations

DSC: Dyadic Sexual Communication Scale
PEPP: Psychosexual Educational Partners Program
PROMIS SexFS: PROMIS Sexual Function and Satisfaction version 2.0
RDAS: Revised Dyadic Adjustment Scale
REDCap: Research Electronic Data Capture

## Data Availability

The datasets generated or analyzed during this study are not publicly available due to the small sample size but are available from the corresponding author on reasonable request.
