# Supplementary material for: A Sexual Health Self-Management Intervention (Psychosexual Educational Partners Program) for Couples With a History of Breast and Gynecological Cancer: Mixed Methods Feasibility Study
Source: JMIR Form Res. 2025 Oct 21;9:e75743. doi: 10.2196/75743 (PMC12587013; doi:10.2196/75743)
Supplement: Multimedia Appendix 1 [file formative_v9i1e75743_app1.docx]

## Participant Experience, Descriptive Summaries

### Length of the Intervention

Seven couples (n=14) participated in the end-of-study qualitative interview. The first 2 questions related to the length of the study and workbook. Thirteen of 14 responded with 7 reporting the 6-week timeframe was just right (54%), 5 reporting it was too short (38%), and 1 (8%) reporting it was too long. Regarding the amount of material, no one felt it was too short, 6 (43%) felt it was just right, and 8 felt it was too long (57%), but 6 of those were referring to Chapter 2 specifically.

### What they liked or didn’t like about the intervention

When asked what they liked and didn’t like about the workbook, 8 out of 14 people mentioned it was a helpful guide for discussions; for example, participants said it gave them structure to follow, ensuring breaks or space for both partners to talk and prompts that raised important topics that might otherwise be avoided. The tone was described as reassuring, affirming and effective at normalizing difficult experiences. Regarding preferences to change the material, each of the following was mentioned once: a photo was disliked, the wording used to introduce a checklist was perceived as insensitive, the word “cancer” was used in a mindfulness activity and was reported to be an unpleasant surprise and one participant recommended adding more medical advice related to symptoms.

To support couples as they moved through the workbook, bi-weekly phone calls were held with study staff and the female cancer survivor. All 7 couples described the calls as important, using descriptors like helpful with accountability and reflection, motivating, and good reminder to stay on track.

Eight out of 14 participants (57%) cited specific aspects of the mindfulness activities they found beneficial. For instance, one participant said the mindfulness activities helped them realize that it was good to make more quiet time for each other without any tasks that needed to be completed. Another appreciated these activities as a break from the focus on sex. Some barriers that were mentioned at least once included time needed to learn (n=1), time needed to practice/couldn’t be rushed (n=1) and did not enjoy/resonate (n=2).

Regarding the activities in general, 10 out of 14 participants (71%) said they were explained well. Additional descriptors included useful, meaningful, challenging, helpful, appropriate, and flowed together well and good variety. Four of the 14 participants (29%) specifically mentioned the value of the weekly check-in activities. For example, one partner stated, *“… with the workbook… you have that … secondary feature to kind of remind you and prompt you which was nice. So it's not always her asking me to sit down and talk to her.”*

### Comfort with using the intervention

Participants were asked to describe their comfort level using the workbook. Reponses ranged from anxious and nervous at the beginning to very comfortable with all of it by the end. Some couples described improvement, but said they still were not fully comfortable discussing sexual problems at the end. Others described an increase in comfort, but possibly only within the context of the workbook, because as one partner put it, the workbook *“gives couples the permission to talk about this and people need permission.”* Another cancer survivor participant explained that the workbook helped her identify the feelings she was having. She explained “they were unpleasant and uncomfortable, and I didn't really want to own that I was having those feelings. (The workbook) made me stop and really kind of say, okay, what's going on here and once you think about it, and you can put words to it, you can talk about it.”

### How the intervention helped

When asked what has the PEPP intervention helped you accomplish, participants provided a variety of insights relating to both emotional and physical intimacy. Responses included setting aside time to talk and be together, being super transparent with each other about difficulties, scheduling meaningful time together, and accepting that things are always changing. One survivor pointed out that the workbook made other things possible because it created accessibility, explaining that she was referred to a therapist who specialized in sexual health and when she called, she was told the waitlist was 18 months long.
